# How can primary care be secured in the long term? – a qualitative study from the perspective of general practitioners in Germany

**DOI:** 10.1080/13814788.2023.2223928

**Published:** 2023-06-30

**Authors:** Julian Wangler, Michael Jansky

**Affiliations:** Centre for General and Geriatric Medicine, University Medical Centre Mainz, Mainz, Germany

**Keywords:** GP shortage, rural physician, country practice, primary care, established care

## Abstract

**Background:**

Securing primary care is an important issue for health policy. Given a threatened shortage of GPs in Germany, there are discussions about what actions to take to guarantee primary care.

**Objectives:**

The aim was to obtain opinions of German GPs towards (a) the status quo and development of primary care, (b) favoured actions to secure it and (c) assessment of the actions taken.

**Methods:**

In 2021 and 2022, 96 semi-structured interviews (criterion sampling) amongst GPs were conducted in all German federal states (41 face-to-face, 32 by telephone, 23 *via* telecommunication application). The data was analysed according to qualitative content analysis. Additionally, a short questionnaire recorded the problem of GP shortage.

**Results:**

Many interviewees fear a veritable shortage of GPs in the future. They identify structural problems linked to the health care system. The interviewees suggested creating a primary care physician system or upgrading the GP position. They proposed greater support of interests about general practice in education and training, a restructuring of curricula and admissions criteria in higher medical education and reforming GP training. Building up multi-professional outpatient care centres and strengthening task shifting are valuable. The interviewees have observed progress in ensuring primary care but see a need for further action.

**Conclusion:**

The study has shown that GPs, from their perspective and experience, make specific suggestions to ensure primary care in the long term. Consequently, it is advisable to consider their points of view when planning, implementing and adjusting steps to strengthen primary care.

KEY MESSAGES

- We explored which approaches GPs propose to secure primary care.
- GPs favour creating a primary care physician system or upgrading the GP position They also proposed greater support of interests with regard to general practice in education and training.
- It seems recommendable to include GPs when planning and implementing actions to strengthen primary care.

## Introduction

In Germany, GPs are the central pillar of outpatient care since most people first go to their GP when they have a health problem, although there is no obligation to do so. When needing sophisticated diagnostics or specialised treatment, GPs usually refer patients to an appropriate specialist practice or other healthcare providers. In many other countries, GPs have a similar role [1].

For some years, securing primary care has been an essential issue for health policy [2]. In Germany, every third GP currently working is 60 years old or older [3]. Consequently, in a European comparison, Germany heads the countries with a rapidly ageing medical profession [4]. According to the Federal Association for Statutory Health Insurance Physicians, in 2020, there were about 4,200 vacancies for GPs nationwide on approximately 45,000 GPs in total [3]. According to calculations, an annual loss of about 1700 GPs compares with an inflow of specialist medical qualifications in primary care of about 1300 per year [5,6]. This is also related to the fact that many young GPs increasingly prefer part-time models and avoid the risk of self-employment [7]. As a result, by 2025, there may be a lack of about 20,000 GPs, especially in rural districts [6,8].

Therefore, there are continuing discussions about the actions to take to secure the provision of primary care. In this article, this means creating structural conditions that ensure a constant flow of (young) GPs moving into the health care system so that the number of available GPs can be stabilised [2,4,5]. Over time, various approaches have been proposed or partially initially implemented [9].

One conceptual focal point targets a structural strengthening of primary care such as in the form of multi-professional outpatient centres or by introducing a primary care physician system, which could make general practice more attractive as a profession [10,11]. In a primary care physician system, every health-insured person registers with a GP who exclusively regulates access to specialists [1]. In addition, new task-shifting models could relieve GPs and make provision more effective [12]. Strategies have also been pursued to set up GPs in rural and structurally-weak areas. These actions include start-up aids, stipends or structured training [5,8,13,14]. A further focus relates to creating a broader recruitment basis for GPs. There are proposals to set quotas for specialist training or to promote lateral entry (recognition of equivalent medical training in specialist areas based on medical training regulations) [15]. Beyond the introduction of rural physician quotas, there are proposals to revise curricula in higher medical education and to change admissions criteria [16]. In addition, expert opinions suggest changes in specialist medical training and its consistent alignment with practical general practice [14].

There has not yet been any work that robustly investigates which approaches GPs propose to secure primary care in the long term structurally and how they assess the efforts made so far [1,16].

The present study aimed to obtain the opinions and attitudes of GPs towards the issues outlined. The main focus was on the following questions:

- How do GPs perceive the status quo and the future development of primary care?
- Which actions do GPs consider to be promising in order to secure primary care?
- How satisfied are GPs with the efforts that have been made so far?

## Methods

### Study design

To investigate the topic at hand, we pursued an exploratory approach using qualitative semi-structured interviews.

### Interview guide

The interview guide was developed using the relevant literature [inter alia 1,5,10,15] (Appendix A). Particularly, the work of van den Bussche [1], who discussed problems concerning ensuring primary care in a bundle, was used. On this basis, it was possible to create a list of measures to be queried. The interview guide was pretested with a small sample of participating GPs and focussed on:

- Status quo and future development of primary care
- Conditions and favoured actions to structurally stabilise and secure primary care
- Assessment of the actions taken so far to secure primary care

Additionally, a one-page short questionnaire was given to all interviewees after the interview. It concentrated on recording the perceived problem of the (threatened) shortage of GPs. With this short questionnaire, we wanted to supplement the qualitative findings and underpin the examined problem quantitatively. In this respect, the overall study is strictly a mixed-methods approach.

### Recruitment and participants

Initially, a pool of 850 contact addresses was compiled using the doctor finder search engine software made available by the Association of Statutory Health Insurance Physicians (Kassenärztliche Vereinigung), which contains a wide variety of GPs in all federal states. We intended to guarantee that (a) for the large states all government districts are represented, (b) single and group practices are equally included if possible and (c) different practice environments are represented (rural community, small town, medium-sized town, large city).

In the course of the criterion sampling method we chose, we also intended that every state would be equally represented in the study regardless of its population size to depict rural regions in particular better. When selecting the advised number of six GPs for each state, the following access criteria applied: at least one group practice, balanced gender relationship, balanced relationship between urban and rural community/small town practices and consideration of older and younger physicians.

In total, 121 general practices throughout Germany were contacted based on the considerations presented above. Those showing interest in taking part were provided with the study information. The period of recruitment extended between May and August 2021. Ultimately, it was possible to acquire 96 GPs for individual interviews.

The interviews took place between July 2021 and April 2022 and were conducted by both authors (both general practice researchers), each running half of the interviews (40–60 min each). It was possible to conduct the interview either verbally in person, by telephone or *via* a telecommunication application. Audio recordings were made of the interviews.

Forty-one interviews were conducted face-to-face, 32 by telephone and 23 via telecommunication application.

### Data analysis

According to Mayring (MAXQDA software), the first author evaluated the transcripts using content analysis [17]. This first entailed pinpointing the key statements, followed by further abstraction and summarisation, finally leading to a categorised system closely based on the interview guide and repeatedly reviewed and modified as necessary during evaluation. At the same time, we gave room to information expressed by the interviewees that differed from our input. Our focus lay on forming logical categories from the various opinions and experiences.

We had set the prior condition that all 96 interviews were to be conducted, regardless of whether indications of a theoretical saturation would occur beforehand.

## Results

### Sample

The sample obtained consisted of the following:

- Form of practice: 42 group practices, 54 single practices
- Practice environment: 30 rural communities/small towns, 36 medium-sized towns and 30 large cities
- Status: 64 practice owners, 32 employed physicians
- Age: on average 54 years old (range: 22 years)
- Gender: 48 male, 48 female

### Status quo and future development of primary care

Almost all interviewed articulated how much they enjoyed and gained fulfilment from their professional life, highlighting the importance of general practice. With regard to the profession, in times of increasing disciplinary specialisation, they perceive a *‘big opportunity for general practice’* (I-56f). If effective actions could strengthen the GP profession, an *‘expert all-round doctor’* (I-19f) would meet *‘considerable demand’* in an ever-more differentiated healthcare system (I-25m). The general practice could *‘gain clout in the next 10–20 years’* thanks to careful modernisation, digitalisation and division of labour (I-48f).

Despite a fundamental conviction *‘that there will always be an important place for the GP’* (I-50f), numerous interviewees expressed *‘serious doubts’* (I-55f). They assumed *‘there could be a veritable shortage of non-hospital general practitioners in an interim phase, let’s say in the next 15 or 20 years’* (I-54m). As the evaluation of the supplementary questionnaire shows, only 26% of the interviewees stated that they believe primary care in Germany will be secure in the upcoming decades (51% tend to say not so well secured, 33% not secured at all).

*If I had to summarise, I’d say the big change – a negative change, unfortunately – is that GPs are dying out. (I-25m)*

The reasons for the decline of primary care physicians are described as complex so that it is *‘not easy to give a quick answer here’* (I-25m).

*Health policy has been working on this topic for decades. So far, no convincing solution has been laid out. (I-71f)*

Older interviewees in rural regions reported severe problems when looking for successors for their practices. Other interviewees were aware of this problem. Thus, in the short questionnaire, 37% stated their practice environment had been affected by a (relatively) substantial decline in general practices (29% less strongly affected, 34% not affected at all). In the coming 10–20 years, 58% of the interviewees expect a (worse) shortage of GPs in rural and structurally-weak areas; 33% even expect a considerable comprehensive lack of GPs.

One significant difficulty is that, despite all the health policies and other actions taken so far, *‘general practice is seen to be insufficiently attractive by young physicians’* (I-44m). The interviewees consider this not a genuine problem for the speciality itself, which offers a variety of development opportunities, but primarily a problem in education and training, as well as regarding the position of primary care in the healthcare system.

*What we must recognise here is that GPs are far too often the stopgaps, who have to jump into the breach for everything. With their time, energy and budget. What sort of image does that generate in public? (I-29m)*

Ergo, most interviewees identified an *‘image problem’* (I-23m) that *‘produces a little incentive to enter this field’* (I-19f). More than half the sample attested to a current falling or too little attractiveness of general practice for young physicians. Regardless, almost all those interviewed would recommend becoming GPs to medical students.

*The profession is great and fulfilling. But it’s not about us, about the insiders. It’s about generating something like a magnetism for the job. And that’s lacking. (I-42m)*

What is noticeable is that – beyond budgetary aspects – many interviewees spoke about an unregulated, partly chaotic and ineffective division of labour between the sectors, which was experienced as a *‘considerable stumbling block for general practice’* (I-34m). Especially the absence of a primary care physician system complicated GPs’ activities *‘in almost every respect: financially and related to time and resources’* (I-71f).

*GPs should get more decision-making and gatekeeper competence. The health care system has to be turned upside down here. (I-14m)*

The interviewees stated that cooperation, communication and coordination did not work properly, GPs were *‘kept in the dark’* (I-76m)and the *‘navigation behaviour of patients in the health care system’* was *‘often arbitrary’* (I-35f). In addition, GPs were not sufficiently involved in the existing structures, particularly in an inter-professional context. There were issues such as too weak development of task shifting and substitution solutions here.

*It’s often said that we GPs are lone wolves. But in my view the health care system often turns us into solitaries. (I-29m)*

*This structural isolation of GPs is perhaps one of the greatest deficits I see in the German healthcare system. We cannot exploit our potential. […] A lot could be done regarding task shifting and relieving GPs. (I-87f)*

Another problem raised relates to the dimension of education and training. From the point of view of several interviewees, prospective physicians who can imagine a GP career were *‘scared off from GP self-employment due to a lack of preparation’* (I-54m). Important factors of uncertainty included questions such as self-employment and practice management. Furthermore, previous medical studies and also part of the training did *‘not really whet the appetite for […] or provide insights in the world of the GP’* (I-48f) because the importance and assets of general practice were not always evident. Some interviewees stated it had been assumed for too long *‘that some students would automatically become GPs’* (I-15f) instead of *‘keeping an eye out and providing encouragement’* during higher medical education and courses of studies and training (I-76m).

Another focal point of criticism is that policy had treated GPs *‘as outdated, conservative people for too long’* (I-19m) and that it had not been anticipated that the following generations would have different conceptions of employment and compatibility. From the point of view of these respondents, there was only a slow and gradual rethinking to address other working time models and a lower desire for self-employment or interdisciplinary connection.

### Favoured actions to secure primary care

Table 1 shows an overview of the approaches and concepts that in the interviewees’ view, would make more or less effective contributions to securing primary care. It can be recognised that measures are favoured to upgrade the position of general practice and adapt education and training to current requirements.

**Table 1.** Agreement with actions to secure primary care.

Proposed action	Number of mentions	Assessment of the benefits of the action (related to the distribution in the sample)
Strengthening and making the position of general practice more effective (especially by introducing a primary care physician system)	49	Very effective
Longitudinal accompaniment of (clinical) medical studies with additional general practice modules	47	Very effective
Restructuring the contents of medical curricula	46	Very effective
Greater changes in the admission criteria for studying medicine (e.g. personality, previous experience)	41	Somewhat effective
Fundamental reform of general practice training	39	Somewhat effective
Significantly increasing the proportion of general medicine in training	37	Somewhat effective
Increased expansion of multi-professional centres of outpatient (primary) care	35	Somewhat effective
Delegation and increased use of non-doctor medical professions	35	Limited effectiveness
Binding definition of the GP catalogue of services	34	Limited effectiveness
(Stronger) requirements planning with targeted regional distribution effects	32	Limited to low effectiveness
Effective recruitment of physicians with the help of incentives and bonuses	29	Limited to low effectiveness
Setting quotas for access to specialist training (see also: Significantly increasing the proportion of general medicine in training)	27	Limited to low effectiveness
Setting up a continuous, nationwide quota for country physicians (clearly regulated for all states, as an on-top quota if necessary)	25	Limited to low effectiveness
Fundamental upgrading of GP remuneration	24	Limited to low effectiveness
Greater and more systematic use of digitisation and telemedicine (including video consultations, prescribing health apps for patient self-management).	23	Low effectiveness
Opening up authorisation for GP activities more for career changers with other disciplinary backgrounds	21	Low effectiveness
Many more student places in medicine	20	Low effectiveness

*General practice has to become much more important in the material in higher medical education. (I-56f)*

*In my eyes, the training is not yet close enough to GP reality and it could also be made more compact. (I-29m)*

The number of interviewees who proposed stablishing a primary care physician system is exceptionally high. This is often combined with the demand for a stronger obligingness of the general practitioner service catalogue.

*The options for action and the portfolio of GP services must be more clearly defined. On the one hand, this will allow the role of the GP to be strengthened. On the other hand, you would have to guarantee the spectrum of services in training. For me, it would be conceivable to no longer link the authorisation for general practice to the specialising in general practice. (I-49f)*

In addition to changed admission criteria or a more extensive restructuring of the content of higher medical education, the interviewees advocated interventions in the process of courses of studies (accompanying longitudinal programmes). Those will enable students to experience general practice work plastically, remove uncertainties and practice diagnostic abilities.

*The people who become GPs later are special. You must start looking for these people as early as possible, e.g. during admission to higher medical education – even beyond any rural physician quotas. (I-64f)*

*I’ve seen it from my own experience as a teaching doctor. An accompaniment programme like this will open up horizons and provide a careful introduction and motivation. (I-33m)*

Other focal points are strengthening training and the (enhanced) expansion of multi-professional care centres that use interdisciplinary networking strategies and could compensate for the loss of a comprehensive GPs network.

*These combined establishments may be a departure from the classic practice model. But they offer the chance for a fundamental modernisation and interdisciplinarity of general practice and will take account of the changed conceptions of the younger generation, of course. (I-19m)*

Those interviewed had comparatively little trust in actions such as rural physician quotas, budget upgrades or digitisation to help general practice in the longer term.

*That [rural physician quotas] can contribute in the medium-term. But I ask myself how much sense these straightjackets make for budding physicians. (I-43f)*

It was possible to state the most favoured action in the brief questionnaire, 45% of interviewees favoured a primary care physician system, followed by different aspects targeted at reshaping education and training.

### Assessment of the actions taken so far

Asked about their general impression of how they assess the actions taken to date to secure general practice, the general conclusion of most interviewees ranged from somewhat reserved to sceptical. Despite first successes such as rising specialist recognition figures, the predominant broad assessment was that greater efforts were required. In the brief questionnaire, 32% stated that the previous (health-policy) efforts to secure primary care were sufficient to achieve this target; 68% were sceptical.

The actions taken to date were frequently criticised as *‘pointing in the right direction, but much too half-hearted’* (I-35f). For example, there was criticism that many states have not specified any on-top quotas for the partially established rural physician quota but have stipulated a proportion of existing higher-education places for this. The interviewees also criticised that not all federal states pursue the rural physician quota and that its arrangement is inconsistent.

Further, reforming human medical curricula of studies initiated with the Master Plan for medical studies 2020 was *‘not a fundamental turning point but a makeshift solution at best’* (I-19m). While *‘efforts to reform are recognisable at least’* about higher medical education, in the area of training there were still *‘too strong forces of inertia’* (I-19m). Some interviewees consider the competence centres for specialist training valuable since they provide targeted training support.

*I think what’s being done there is very clever but these institutions must be given more support and clout. (I-57m)*

## Discussion

### Main findings

Despite a fundamental conviction towards the importance of the GP profession, many interviewees expressed concern that there could be a veritable shortage of GPs in the outpatient sector in the coming years. From their standpoint, the main problems were primarily seen as structural problems linked to the German health care system and also in education and training. Besides, the ones interviewed perceived deficits in (regional) needs planning and promoting incentives.

The interviewees suggested creating a primary care physician system and an upgraded role for GPs. Stronger promotion of the interests with regard to general practice in education and training was also considered to be sensible, as were restructuring curricula and the admissions criteria to study medicine, as well as a reform and enhancement of general practice training. Besides, they propose a stronger transfer of classic solo practices of GPs into multi-professional centres.

When it comes to ensuring primary care, the interviewees have observed a certain amount of progress but see a need for further action. Although the interviewees judge some of the measures taken as important, their implementation is sometimes inconsistent (e.g. rural physician quota, reform of human medical curricula).

### Comparison with existing literature

Numerous works have been published warning of a significant shortage of primary care providers in the coming decades [2–8]. As Contandriopoulus et al. work out in their meta-analysis [18], evidence suggests that public healthcare systems must change significantly to preserve their capacity to maintain universal access to healthcare. However, while many expert opinions on the healthcare system have been developed, there is a lack of inclusion of the GP perspective. Accordingly, hardly any studies determine how GPs view the problem of a threatened shortage of primary care providers from their own experience and which solutions they consider appropriate to ensure primary care in the long term [1,16].

The results of the present study show overlaps with the proposals made by health services experts towards certain concepts to secure primary care [19]. For example, as well as many of those interviewed, van den Bussche puts solution approaches such as establishing a primary care physician system at the centre of his suggestions – a point called for by the German College of General Practitioners and Family Physicians (DEGAM) for years [1]. In its position paper it details that not only will this lead to an improvement in overall care but also an effective ‘improvement in the attractiveness of the GP profession’ as an important argument [11]. A further complex that unites the interviewed physicians with what the experts are calling for relates to the binding definition of a GP catalogue of services, the establishment of multi-professional care centres (chance for synergies with cross-sector solutions) [6,17,20], strengthening task shifting (use of non-doctor medical professions) and also greater regulation of access to specialist training [1,12,16,21]. According to Contandriopoulus et al. capacity strengthening in primary care provision is widely considered to be an approach with the potential to expand the interdisciplinary composition of primary care teams [18], the scope of practice of non-physician team members and their intersectoral action.

At the same time, it can be recognised from the interviews that GPs favour approaches that are not always the focal point of common expertise. For example, they place considerable value on longitudinal interventions during higher education and believe, partly from their own experience, these are particularly effective in anchoring early interest in general practice among students and communicating specific knowledge of non-hospital activities. The longitudinal lesson through general practice is a component in the German Master Plan for medical studies 2020. The expert committee’s report also highlighted the importance of additionally anchored ranges in general medical courses of studies (voluntary rural physician tracks, enhanced introductions to professional fields etc.) [22]. International studies have demonstrated that completing general practice block internships, for example, significantly influences the later choice of profession or the readiness to take up non-hospital work [23,24].

Many GPs emphasise that new work and employment models should be developed for young colleagues, for example, by transferring the classic single-doctor general practice concept into new, more integrated settings [10,25,26]. This is covered by von Huenges et al. van den Bussche et al. who surveyed physicians in training [1,14,27,28]. As was also seen in the interview results, the studies in question showed a need for further-reaching reform with regard to duration, contents and didactics. Competence centres connected to universities, which the expert committee has also suggested as comprehensive establishments [19], can support training and strengthen young GPs through structured course programmes, mentoring and building training associations [16].

It is noticeable that the solution approaches pursued by German health policy in the past few years, such as rural physician quotas, were only seen as helpful to a limited degree by the interviewees. One reason seems to be that GPs want long-term actions that create motivation and promote the image of general practice. They also criticise the different arrangement of rural physician quotas that depend on the state and its qualitative arrangement. In contrast to the expert opinions submitted the interviewees are reserved regarding admitting more lateral entrants to general practice and lowering the barriers for training. Concern about the erosion of quality standards, essential for general practice, covers the positions formulated by the DEGAM once again [1,11].

Overall, it seems recommendable to consistently include GPs and their experience when planning, implementing and evaluating actions to counter the (threatened) shortage of primary care providers [29]. Their professional group is in the minority in medical and scientific committees and should be considered more by political decision-makers. It would also be conceivable that closer coordination with the health policies of the states and municipalities would result from the professional organisation here.

### Strengths and limitations

The authors consider it a strength of the present work that it can be seen as a supplement to existing studies that have dealt with career paths and career breaks of budding medicks during their studies and training.

Despite the efforts to recruit a heterogeneous sample of GPs, the study cannot claim to be fully representative. It is also possible that physicians interested in the issue were more motivated to participate in the interviews.

The transcription, coding and categorisation were – not as usual in qualitative research – carried out by only one assessor, as it was impossible to include both authors due to time and workload. Nevertheless, to be as objective and open to new codes as possible in the coding process, the assessor followed Mayring’s principles of intracoder reliability [17]. This means that samples of the data material were coded several times. In this way, whether the text passages were assigned to the same category was checked.

Besides, telephone and online interviews may have led to a lower willingness for interviewees to provide information and personal insights than face-to-face interviews.

## Conclusion

Many interviewees expressed concern that there could be a veritable shortage of GPs in the coming years. The main problems were primarily structural problems linked with the German healthcare system. The GPs suggested creating a primary care physician system and an upgraded role for GPs as well as a more vigorous promotion of primary care in education and training, a reform of general practice training and a transfer of classic solo practices of GPs into multi-professional centres. Regarding ensuring primary care, the interviewees see a need for further action.

The study has shown that GPs, from their own perspective and professional experience, make specific suggestions to ensure primary care in the long term. Consequently, it is advisable to consider their points of view when planning, implementing and adjusting steps to strengthen primary care.

## Data Availability

The datasets generated and/or analysed during the current study are not publicly available beacause participants did not give permission for recordings or transcripts to be released to other researchers but are available from the corresponding author at reasonable request.

## Appendix A

### Interview guide

Please try to imagine and describe the following: How do you see the future of GP care in 10 to 20 years?

Which significant changes do you see? What are these changes related to?

Which challenges will the ‘GP’ profession face and what are the opportunities?

To what extent are you currently observing more of a rising or more falling attractiveness of general practice for young GPs, if you compare this with the situation a few years ago? What do you think are the reasons for this?

To what extent would you recommend young medical students today to become GPs and to what extent would you advise them against this? Why?

Suppose we think about guaranteeing GP care more long term. Do you see the future with confidence or are you worried? What are your reasons for this?

In your assessment or your experience, how far do you think general practice care is secured in the upcoming decades? Why (not)?

In your view, where are the most significant problems?

In your view, which conditions and actions would be auspicious and should be implemented as a priority to guarantee general practice care long-term?

How satisfied are you with the efforts and actions taken so far to secure general practice care? What do you think is positive or negative?

In your opinion, which efforts should be reinforced or changed?

I’d now like to name some different actions. Please tell me how much sense or effectiveness you think these are for securing general practice care. Try to justify your position if possible.

- **Introduction of a primary doctor system** that makes GPs the first and binding contact for patients and prevents them from going to specialists directly and in parallel. (Advocates of the primary doctor system do not argue that there will be too much, too little or the wrong care, but that there will also be an upgrading of the decision-making expertise, financial support and an image gain for general practice care.)
- **Setting a binding catalogue of GP services** to clearly define the portfolio of GP tasks and avoid any overburdening of GPs (e.g. by ensuring sufficient qualifications and working hours).
- **Moving away from classic practice models to multi-professional centres for outpatient (primary) care** to expand general practice care (e.g. health centres near hospitals or in urban centres that allow multi-professional cooperation and other working models).
- **Delegation and increased use of non-doctor health professions** as well as expanding their competences
- Significantly **increasing the proportion of general medicine in training** (e.g. by one-third)
- Opening up **authorisation for GP activities** more for career changers with other disciplinary backgrounds
- **Setting quotas for access to specialist training**
- **(Stronger) requirements planning with targeted regional distribution effects**
- **Effective doctor staff recruitment** (increased work with incentives and rewards, e.g. from municipalities and subsidies and bonuses, such as when someone locates in a rural area)
- **Fundamental reform of general practice training** (including shortening and more flexibility, greater focus on central competencies for GP work)
- **Many more student places in medicine**
- **Setting up a continuous, nationwide quota for country doctors** (clearly regulated for all states, as an on-top quota if necessary)
- **Greater changes to the admission criteria to study medicine** (take greater and more comprehensive account of larger broad factors such as personality and curricular specifics)
- **Curriculum content restructuring of medicine higher education** (better and more targeted preparation for outpatient, non-hospital prospects, in particular, general practice)
- **Fundamental uprating of GP remuneration** (e.g. that this is at the same level as specialists, at least, given the same length of training and longer working time)

Do you have any other ideas to secure general practice medicine long-term?

### Supplementary questionnaire (submitted after the interview)

1. **What would you say? How heavily is the area in which you have your GP practice affected by a disappearance of GP care or a decline in GP practices?**
    ○ Very heavily affected  ○ Somewhat heavily affected ○ Less heavily affected  ○ Not affected at all ○ Difficult to say, don not know
2. **What is your assessment? How well is general practice care in Germany secured for future decades?**
    ○ Very well secured ○ Somewhat well secured  ○ Not so well secured ○ Not secured at all
    ○ Difficult to say, don’t know
3. **What do you expect from the range of care GPs offer in Germany in the next 10 to 20 years?**
    ○ Significant, widespread shortage of GPs
    ○ (Increased) shortage of GPs in rural and structurally weak regions
    ○ No real shortage of GPs
    ○ Difficult to say, don not know
4. **In the past few years, several attempts have been made to secure general practice care. Do you think the actions taken so far will be sufficient to ensure general practice care or not?**
    ○ Fully sufficient ○ Largely sufficient ○ Only partially sufficient ○ Not sufficient at all
5. **Is there any one action that you consider to be particularly important, effective and urgent to contribute to securing general practice care? If yes, which action would this be?** (Please only state what you consider the most important single point.)
